# The Distribution of Incomplete Gastric Intestinal Metaplasia (GIM) Subtype among Biopsy Sites according to the Updated Sydney System and Its Association with GIM Extension

**DOI:** 10.1155/2018/4938730

**Published:** 2018-05-08

**Authors:** Duc Trong Quach, Huy Minh Le, Trung Sao Nguyen, Toru Hiyama

**Affiliations:** ^1^Department of Internal Medicine, University of Medicine and Pharmacy at Ho Chi Minh City, Ho Chi Minh City, Vietnam; ^2^Department of Gastroenterology, Gia Dinh People's Hospital, Ho Chi Minh City, Vietnam; ^3^Department of Surgical Pathology, University of Medicine and Pharmacy at Ho Chi Minh City, Ho Chi Minh City, Vietnam; ^4^Health Service Center, Hiroshima University, Higashihiroshima, Japan

## Abstract

**Background:**

Current guidelines recommend that extensive gastric intestinal metaplasia (GIM) be considered as a high-risk marker for the development of gastric cancer (GC). But there is emerging evidence that the incomplete GIM subtype is also a high-risk marker.

**Aims:**

To evaluate the performance of biopsy sites according to the updated Sydney system on detecting the incomplete GIM subtype and to assess its association with GIM extension.

**Patients and methods:**

A cross-sectional study was conducted on 280 Vietnamese patients with nonulcer dyspepsia. Biopsy specimens were taken from gastric sites according to the updated Sydney system, and sections were routinely stained with Giemsa and hematoxylin and eosin. Biopsy specimens with intestinalization were further evaluated for GIM subtypes with alcian blue 2.5 and periodic acid Schiff stainings. Two experienced pathologists jointly examined all the specimens and reached consensus.

**Results:**

The rates of patients with GIM and the incomplete GIM subtype were 81 (28.9%) and 24 (8.4%), respectively. There was no GIM in specimens taken from the greater curvature of corpus. The proportions of the incomplete GIM subtype detected at the incisura angularis, lesser curvature of corpus, lesser curvature of antrum, and greater curvature of antrum were 34.3% (12/35), 34.5% (10/29), 40.5% (17/42), and 31.6 (6/19), respectively, which were not significantly different (*p* = 0.89). The presence of an incomplete GIM subtype was associated with multifocal GIM (*i.e.*, ≥3 out of 5 biopsy sites with GIM) (OR = 4.02, CI 95%, 1.33–12.16, *p* = 0.022) and extensive GIM (*i.e.*, GIM in specimens from both of corpus and antrum) (OR = 2.89, CI 95% 1.04–8.02, *p* = 0.045).

**Conclusions:**

The proportions of an incomplete GIM subtype were not significantly different among gastric biopsy sites with intestinalization. The association between an incomplete GIM subtype and GIM extension, therefore, may be due to an sum accumulation effect.

## 1. Introduction

Gastric intestinal metaplasia (GIM) is a precancerous lesion in the decades-long multistep process from gastritis to gastric cancer (GC) [1]. Recent population-based studies reported that the incidence rate of GC in patients with GIM was about 0.38 to 1.29 per 1000 person years [2, 3]. Therefore, surveillance of patients with GIM is required to detect GC in the early stage. This is especially important in the era of therapeutic endoscopy, so as to reduce morbidity and mortality of patients with GC. However, the worldwide prevalence of GIM is significantly high. In the general population, the prevalence of GIM was 25% (19–30%) [4]. As a consequence, a cost-effective approach should identify patients with high-risk GIM characteristics as candidates for surveillance. Current guidelines recommend performing mapping biopsies according to the updated Sydney protocol in patients with gastritis and considering extensive GIM as a high-risk marker for GC [5, 6]. Although routine GIM subtyping is not recommended, recent studies suggest that an incomplete GIM subtype is also an important high-risk marker [7–9]. However, the performance of biopsy sites according to the updated Sydney system to detect this GIM subtype has not been evaluated. This study aims at assessing the distribution of GIM subtypes among gastric biopsy sites according to the updated Sydney protocol and its association with GIM extension.

## 2. Patients and Methods

### 2.1. Patients

This cross-sectional study was conducted based on data from two hundred and eighty patients with nonulcer dyspepsia who were enrolled with a predefined protocol at the University Medical Center of Ho Chi Minh City, Vietnam, between March 2008 and April 2009. The patients gave informed consent and information regarding familial history of GC and smoking status. Exclusion criteria were history of gastric resection, taking antibiotics or proton-pump inhibitors within 4 weeks, and having reflux esophagitis, gastroduodenal ulcers, or endoscopic lesions suspected of GC. The protocol was approved by the ethics committee of the University of Medicine and Pharmacy at Ho Chi Minh City, Vietnam.

### 2.2. Endoscopy Examination

One experienced endoscopist (DTQ) performed all endoscopic examinations. Olympus videoscopes with conventional white light (model GIF-160; Olympus, Tokyo, Japan) were used. Six specimens were taken from each patient. Five specimens for histopathological examination were taken according to the updated Sydney system and were sent to the Department of Surgical Pathology, University Medical Center of Ho Chi Minh City, in different jars according to biopsy sites [10]. The sixth specimen used for the rapid urease test was taken from the greater curvature of antrum.

### 2.3. Pathological Examination

**Figure 1:**
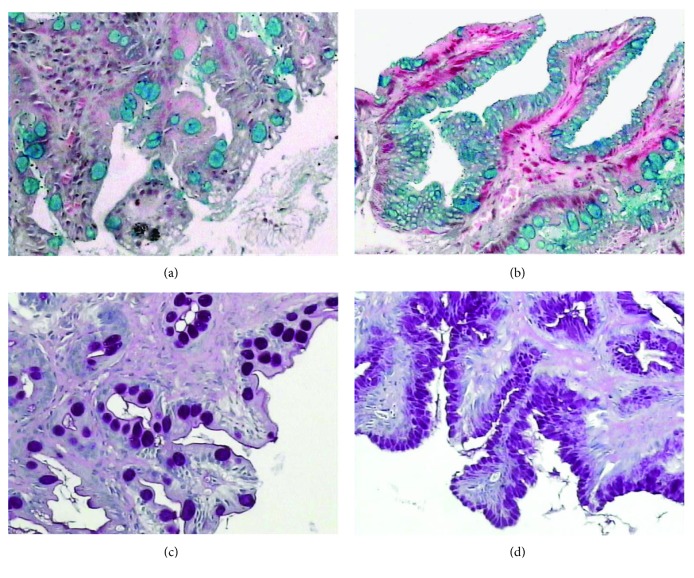
(a, b) Complete GIM subtype characterized by goblet cells stained blue or red by alcian blue (pH 2.5) and PAS stain, respectively, and the presence of absorptive nongoblet cells, magnification ×100. (c, d) The incomplete GIM subtype characterized by the presence of goblet cells and mucin-secreting columnar cells staining blue and red by alcian blue (pH 2.5) and PAS, respectively, magnification ×100.

Biopsy samples were fixed in formalin 10%. Sections were cut at 5 *μ*m and stained with Giemsa and hematoxylin and eosin. GIM at each biopsy location was recorded. In addition, sections with GIM were stained with alcian blue (pH 2.5) and periodic acid Schiff (PAS) to identify the GIM subtype. Two experienced pathologists (HML and TSN), blinded to any clinical and endoscopic information, jointly examined all the specimens and reached a consensus. In this study, we arbitrarily defined multifocal GIM as GIM detected at ≥3 biopsy sites, and extensive GIM as GIM detected in both biopsy specimens taken from the corpus and antrum. Complete GIM was defined as the presence of goblet cells without acidic alcian blue (pH 2.5)-PAS-positive material in columnar-type cells. Incomplete GIM was defined as the presence of goblet cells with acidic mucins in goblet and adjacent columnar-appearing cells [11]. Dysplasia, when present and definite, was graded as low grade or high grade.  Figure 1 shows examples of the complete and incomplete forms of GIM.

### 2.4. *Helicobacter pylori* Diagnosis

The local rapid urease test and histopathologic examination were used to detect *Helicobacter pylori (H. pylori)* infection. Cases were considered *H. pylori* positive if the bacteria were histopathologically detected and/or the local rapid urease test was positive.

### 2.5. Statistical Analysis

Patients in the study were characterized with regard to the most advanced subtype of GIM and were divided into three groups: patients without GIM, patients with only complete GIM, and patients with an incomplete GIM subtype at ≥1 biopsy sites. Continuous variables were expressed as mean ± standard deviation (SD) and confidence interval 95% (CI 95%), respectively. Risk factors for GIM and the incomplete GIM subtype examined in this study included age, sex, smoking status, *H. pylori* infection, and family history of GC. Univariable and multivariable analysis were applied. Odds ratios (ORs) and CI 95% were calculated to assess the strength of association between variables. The Pearson chi-squared test was used to test the difference of incomplete GIM subtype proportions among biopsy sites, between subgroups of patients with/without multifocal and extensive GIM. A two-tailed *p* value < 0.05 was considered as significant. All statistical analyses were performed with SPSS software version 19.0 (SPSS, Chicago, IL, USA).

## 3. Results

**Figure 2:**
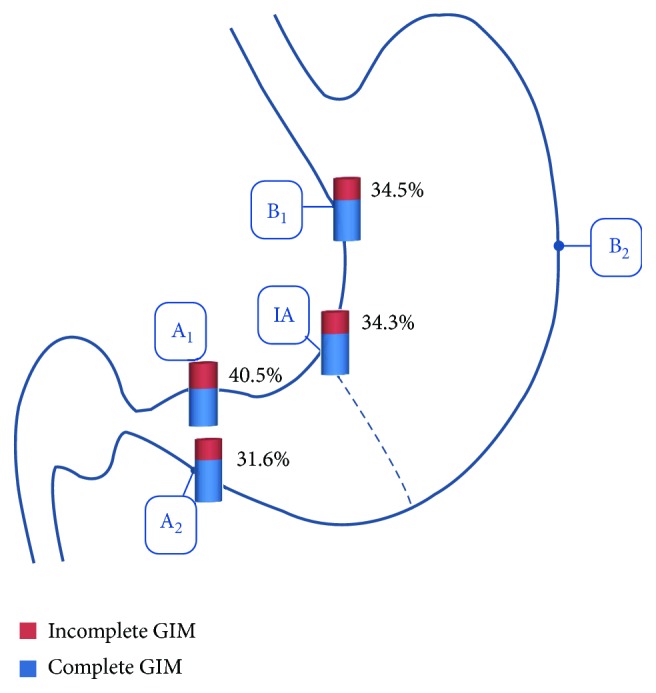
Proportions of the incomplete GIM subtype among biopsy sites with intestinalization (IA: incisura angularis; A1: antrum, lesser curvature; A2: antrum, greater curvature; B1: corpus, lesser curvature; B2: corpus, greater curvature).

**Figure 3:**
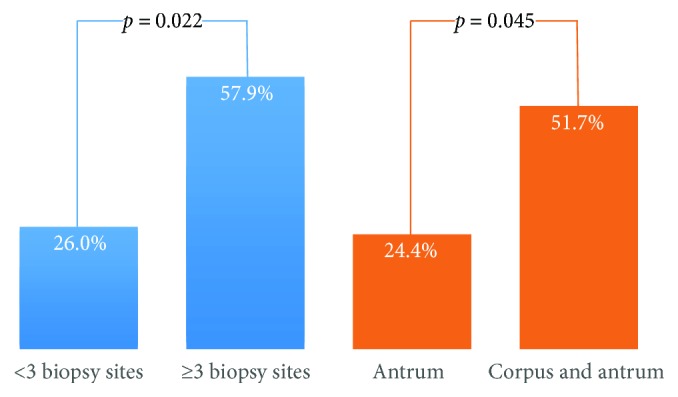
Different rates of the incomplete GIM subtype according to biopsy sites with intestinalization.

The demographic and pathologic characteristics of patients in the present study are presented in Table 1. There were 81 (28.9%) patients with GIM, but there was no GIM in specimens taken from the greater curvature of the upper corpus. The rates of multifocal and extensive GIM were 23.4% (19/81) and 35.8% (29/81), respectively. GIM subtype could not be identified in 12 patients as the specimens affected by GIM were too small for immunohistologic staining. The rates of patients with complete and incomplete GIM subtypes were 55.6% (45/81) and 29.6% (24/81), respectively. In multivariable analysis, age ≥ 40, smoking, and *H. pylori* infection were independent risk factors of an incomplete GIM subtype (OR = 13.87, CI 95% 1.77–108.48, *p* = 0.012; OR = 3.53, CI 95% 1.36–9.14, *p* = 0.009; and OR = 6.35, CI 95% 2.18–14.48, *p* = 0.001), respectively (Table 2). The distribution of GIM subtypes among biopsy sites with intestinalization was not statistically significant (Table 3 and Figure 2). However, there were significant associations between the presence of an incomplete GIM subtype with multifocal GIM (OR = 4.02, CI 95% 1.33–12.16, *p* = 0.022) and extensive GIM (OR = 2.89, CI 95% 1.04–8.02, *p* = 0.045) (Table 4 and Figure 3).

## 4. Discussion

In this study, we only recruited Vietnamese patients with nonulcer dyspepsia. A cohort study in Japan reported that 4.7% (21/445) of patients with nonulcer dyspepsia developed GC within 8 years of follow-up, and baseline GIM was one of the important risk factors [12]. The rate of GIM in our study was 28.9%, which is comparable with data from other countries in the world [4]. The rate of *H. pylori* infection in our study was 49.6%. The infection was an independent risk factor of GIM as well as the incomplete GIM subtype in multivariable analysis with the odds ratios of 3.69 and 6.35, respectively. There was no GIM in biopsy specimens taken from the greater curvature of the upper corpus (site B2) in our study (Figure 2). The explanation is that this site is among the last to manifest GIM in patients with *H. pylori* gastritis [13]. The natural history of *H. pylori* gastritis is for the inflammation to progress from the antrum into the adjacent corpus, resulting in an atrophic front characterized by the replacement of native glands with either pseudopyloric metaplasia and/or GIM. But the proximal progression is more rapidly up the lesser curve than the greater curvature [13]. A previous study comparing GIM among Asian populations showed that GIM scores were significantly lower in *H. pylori*-positive Vietnamese patients than in *H. pylori*-positive Japanese patients. In this study, GIM was also rarely detected in specimens taken from the biopsy site B2 in Vietnamese patients [14].

Although GIM is a risk factor of GC, the risk level is significantly different depending on the distribution of GIM. Extensive GIM is reported as a high-risk marker for GC development [6, 15, 16]. In a case-control study using an extensive biopsy protocol in Colombian patients, Cassaro et al. reported that there were four topographical patterns of intestinalization. The focal and antrum-predominant types were not significantly different between patients with GC and nonulcer dyspepsia, but the “Magenstraße” type (involving the lesser curvature from cardia to pylorus) and the “Diffuse” type (involving essentially the entire gastric mucosa with the exception of the fundus) were associated with higher GC risk (OR = 5.7 and OR = 12.2, resp.) [15]. However, the definition of extensive GIM in the literature is still arbitrary. Reddy et al. defined GIM as extensive if GIM was present in at least two gastric sites or moderate or marked if GIM was noted in at least two biopsy specimens [16]. Cassaro et al. defined GIM as multifocal and extensive if GIM presented in more than 3 and 6 out of 12 biopsy sites, respectively [15]. A recent European guideline defined GIM as multifocal or extensive if GIM was distributed multifocal including the lesser curvature of the corpus and fundus and recommended performing endoscopic surveillance every 3 years [6]. In daily practice, extensive biopsy protocol is not practical and mapping biopsies according to the updated Sydney system is currently recommended for patients with gastritis [5].

The OLGIM (operative link on gastric intestinal metaplasia) assessment has been proposed for the staging of gastritis but it is considered difficult to use in clinical practice, and it has the disadvantage of considerable inter- and intraobserver variation [6]. Recent cohort studies reported that the incomplete GIM subtype was related to the development of GC in different regions, even in countries with a low prevalence of GC [7–9]. Interestingly, Cassaro et al. reported that the presence of the incomplete GIM subtype was associated with multifocal and extensive GIM [15]. The likelihood of detecting the incomplete IM subtype in this study was much lower when GIM was sparse (<4 sites involved) than when it was more extensive (≥4 sites involved out of 12 biopsy sites) (7% versus 60%, *p* < 0.001). In our study, we defined GIM as multifocal or extensive if GIM was detected in ≥3 sites or in both specimens from the antrum and corpus, respectively. We also found that there were significant associations between the presence of the incomplete GIM subtype and extensive GIM (OR = 2.89, CI 95% 1.04–8.02) and multifocal GIM (OR = 4.02, CI 95% 1.33–12.16). The performance of biopsy sites according to the updated Sydney system in detecting the incomplete GIM subtype has not been investigated. Our study showed that this GIM subtype could be identified in all biopsy sites affected with GIM, but its proportion among biopsy sites was not significantly different, accounting for about one third of GIM (Table 3 and Figure 2). This finding suggests that the association between the incomplete GIM subtype and extensive/multifocal GIM is due to a sum accumulation effect. It was also reported that the absence of incomplete GIM in this scenario was not reliable as its distribution was often heterogeneous [17]. Whether patients with both of these risk factors will develop GC more often or faster than patients with only one risk factor is an interesting research theme.

In our study, patients with few biopsy sites with intestinalization still had the incomplete GIM subtype. The proportions of the incomplete GIM subtype in patients with 1 or 2 biopsy sites with intestinalization were 23.5% and 31.3%, respectively. The challenge is whether GIM subtyping is helpful in stratifying risk levels of GC development in these patients. Pittayanon et al. reported that the incomplete GIM subtype but not OLGIM stage was significantly associated with the development of GC in Thailand. Among 10 patients with the incomplete GIM subtype, five patients developed high-grade dysplasia or GC within 4 years. A recent cohort study on 456 patients in Britain provided reliable evidence about the progression of the incomplete GIM subtype to GC during a 16-year follow-up period. Unfortunately, the rates of multifocal and extensive GIM were not addressed in these studies. Therefore, future cohort studies are required to evaluate the rate of multifocal and extensive GIM for GC development.

This study has some limitations. First, this is a single-center study with a small number of patients. Second, the study was conducted in Vietnamese, a population with a moderate prevalence of GC. Therefore, the distribution of the GIM subtype at the upper corpus in greater curvature should be further investigated in high-risk populations. Third, imaging-enhanced endoscopes were not used in this study. If these equipment were used, then the detection rates of GIM would be higher. Nevertheless, the results of this study reflect real-life practice and we think that this issue does not significantly affect the proportion of GIM subtyping based on the mapping biopsy strategy.

In conclusion, our study showed that there was a significant association between the presence of the incomplete GIM subtype and the extent of GIM. As the proportions of the incomplete GIM subtype were not significantly different among gastric biopsy sites with intestinalization, this association might be due to the sum accumulation effect.

## Figures and Tables

**Table 1:** Demographic and pathologic characteristics of patients in the study.

*N*	280
Age (mean ± SD)	46.1 ± 10.5
Sex *(n, %)*	
Male	140 (50)
Female	140 (50)
Smoking *(n, %)*	
Yes (current or ex-smokers)	59 (21.1)
No	221 (78.9)
First-degree relatives with GC *(n, %)*	
Yes	8 (2.9)
No	272 (97.1)
*Helicobacter pylori* infection *(n, %)*	
Positive	139 (49.6)
Negative	141 (50.4)
OLGA gastritis stage *(n, %)*	
0	115 (41)
I	119 (42.5)
II	33 (11.8)
III	8 (2.9)
IV	5 (1.8)
Gastric intestinal metaplasia (GIM) *(n, %)*	
Yes	81 (28.9)
No	199 (71.1)
Number of biopsy sites with intestinalization *(n, %)*	
4	9 (3.2)
3	10 (3.6)
2	17 (6.1)
1	45 (16.0)
0	199 (71.1)
GIM subtype *(n, %)*	81 (28.9)
Complete	45 (55.6)
Incomplete	24 (29.6)
Unidentified	12 (14.8)
Gastric biopsy sites with incomplete GIM detection *(n, %)*	
Antrum (lesser curvature)	17 (70.8)
Incisura angularis	12 (50.0)
Corpus (lesser curvature)	10 (47.7)
Antrum (greater curvature)	6 (25.0)
Corpus (greater curvature)	0
Dysplasia *(n, %)*	
High grade	0
Low grade	7 (2.5)

OLGA: operative link on gastritis assessment gastritis stage, GIM: gastric intestinal metaplasia.

**Table 2:** Risk factors of GIM and the incomplete GIM subtype in univariable and multivariable analysis.

	Gastric intestinal metaplasia (GIM)				Incomplete GIM subtype			
	Univariable analysis		Multivariable analysis		Univariable analysis		Multivariable analysis	
	*p*	OR (CI 95%)	*p*	OR (CI 95%)	*p*	OR (CI 95%)	*p*	OR (CI 95%)
Age ≥ 40	0.001	4.632 (2.109–10.174)	<0.001	6.083 (2.671–13.855)	0.007	9.762 (1.294–73.642)	0.012	13.873 (1.774–108.488)
Sex	0.792	0.901 (0.537–1.511)	**—**	**—**	0.524	0.720 (0.308–1.684)	**—**	**—**
Smoking	1.000	0.993 (0.527–1.872)	**—**	**—**	0.020	2.857 (1.197–6.818)	0.009	3.537 (1.368–9.147)
Family history of GC	0.048	4.298 (1.003–18.428)	0.040	5.500 (1.085–27.883)	0.153	3.621 (0.689–19.022)	**—**	**—**
*H. pylori* infection	0.001	2.896 (1.680–4.993)	<0.001	3.695 (2.068–6.602)	0.002	4.439 (1.606–12.269)	0.001	6.354 (2.184–14.485)

OR: odds ratio, CI: confidence interval.

**Table 3:** The distribution of GIM subtypes according to biopsy site with intestinalization.

Biopsy sites	Total number of patients with successful GIM subtyping	GIM subtype *(n, %)*	
		Incomplete	Complete
Incisura angularis	35	12 (34.3)	23 (65.7)
Corpus (lesser curvature)	29	10 (34.5)	19 (65.5)
Antrum (greater curvature)	19	6 (31.6)	13 (68.4)
Antrum (lesser curvature)	42	17 (40.5)	25 (59.5)

Chi square test: *p* = 0.89.

**Table 4:** The association between multifocal GIM and the incomplete GIM subtype.

Number of biopsy sites with GIM	GIM subtype *(n, %)*		Total
	Incomplete	Complete	
1 site	8 (23.5)	26 (76.5)	34 (100)
2 sites	5 (31.3)	11 (68.7)	16 (100)
3 sites	4 (40.0)	6 (60.0)	10 (100)
4 sites	7 (77.8)	2 (22.2)	9 (100)

Chi square test with Yate's correction, *p* = 0.07.

## Data Availability

The data used to support the findings of this study are available from the corresponding author upon request.
